# Genetic Disruption of the γ-Glutamylcysteine Ligase in PDAC Cells Induces Ferroptosis-Independent Cell Death In Vitro without Affecting In Vivo Tumor Growth

**DOI:** 10.3390/cancers14133154

**Published:** 2022-06-28

**Authors:** Boutaina Daher, Willian Meira, Jerome Durivault, Celia Gotorbe, Jacques Pouyssegur, Milica Vucetic

**Affiliations:** 1 Medical Biology Department, Centre Scientifique de Monaco (CSM), 98000 Monaco, Monaco; boutaina.daher@ibgc.cnrs.fr (B.D.); wmeira@unice.fr (W.M.); jdurivault@centrescientifique.mc (J.D.); cgotorbe@centrescientifique.mc (C.G.); 2 Centre A. Lacassagne, Institute for Research on Cancer & Aging (IRCAN), Université Côte d’Azur, CNRS, INSERM, 061000 Nice, France

**Keywords:** ferroptosis, glutathine, γ-glutamylcysteine ligase, GPx4, lipid hydroperoxides

## Abstract

**Simple Summary:**

The newly described form of iron-dependent cell death, called ferroptosis, has emerged as a powerful strategy for eradicating cancer cells. This is of particular importance for pancreatic ductal adenocarcinoma (PDAC), which has been shown to be one of the most aggressive tumors, with a five-year overall survival of less than 8%. The aim of the present study is to identify the most potent and selective target for the induction of ferroptosis in PDAC cells. The results presented here are of great importance not only for the development of novel and more effective anti-cancer therapeutics, but also anticipate potential resistant mechanisms that cancer cells might deploy. This way, ferroptosis-based therapeutics may be a step ahead of highly adaptable cancer cells.

**Abstract:**

The conceptualization of a novel type of cell death, called ferroptosis, opens new avenues for the development of more efficient anti-cancer therapeutics. In this context, a full understanding of the ferroptotic pathways, the players involved, their precise role, and dispensability is prerequisite. Here, we focused on the importance of glutathione (GSH) for ferroptosis prevention in pancreatic ductal adenocarcinoma (PDAC) cells. We genetically deleted a unique, rate-limiting enzyme for GSH biosynthesis, γ-glutamylcysteine ligase (GCL), which plays a key role in tumor cell proliferation and survival. Surprisingly, although glutathione peroxidase 4 (GPx4) has been described as a guardian of ferroptosis, depletion of its substrate (GSH) led preferentially to apoptotic cell death, while classical ferroptotic markers (lipid hydroperoxides) have not been observed. Furthermore, the sensitivity of PDAC cells to the pharmacological/genetic inhibition of GPx4 revealed GSH dispensability in this context. To the best of our knowledge, this is the first time that the complete dissection of the xCT-GSH-GPx4 axis in PDAC cells has been investigated in great detail. Collectively, our results revealed the necessary role of GSH in the overall redox homeostasis of PDAC cells, as well as the dispensability of this redox-active molecule for a specific, antioxidant branch dedicated to ferroptosis prevention.

## 1. Introduction

Ferroptosis is a regulated type of cell death that occurs as a result of the iron-dependent propagation of a lipid hydroperoxidation event in the plasma membrane of the cell. Studies arguing in favor of the potential of ferroptosis inducers/inhibitors for clinical use in many different human pathologies, including cancer, diabetes, neurodegenerative disorders, ischemia-reperfusion injuries, etc. (reviewed in [1]), have been exponentially growing. In the context of anti-cancer treatment, an increasing amount of data points to two major targets for ferroptosis induction in tumor cells—the cystine-glutamate exchanger (Xc- system, or more precisely, its transporter subunit known as xCT and its chaperon CD98) and the glutathione peroxidase 4 (GPx4), that are, respectively, involved in the maintenance of the cysteine intracellular pool and the removal of the oxidative damages of plasma membrane lipids, preventing ferroptosis [2,3,4]. The link connecting these two pathways is glutathione (GSH), considering that xCT provides the cysteine necessary for the synthesis of GSH [5], while the selenoprotein GPx4 uses it as a reducing power for the removal of lipid hydroperoxides [6,7,8]. 

GSH is a small, non-enzymatic antioxidant composed of three non-essential amino acids. The rate-limiting step in the biosynthesis of GSH is the reaction catalyzed by the enzyme γ-glutamylcysteine ligase (GCL) composed of a catalytic (GCLc) and a modifier subunit (GCLm), in which the dipeptide γ-glutamyl-cysteine (γ-GluCys), with a unique abd high-energy γ-peptide bond, is formed. Full body genetic deletion of the GCLc is lethal in mouse embryos [9], while GCLm knockout mice, although alive, show a significant decrease of tissue GSH levels [10]. The second and ultimate step in this biosynthetic process is catalyzed by the glutamate synthetase (GS), forming the tripeptide γ-glutamyl-cysteinyl-glycine (γ-GluCysGly), also known as GSH. 

Since its conceptualization in 2012 by Dixon and Stockwell [2], ferroptosis has been frequently described as the consequence of GSH depletion leading to the loss of GPx4 activity. Indeed, GSH depletion by pharmacological means (usually by buthionine sulfoximide, BSO) has been reported as, at least partially, an inducer of ferroptosis in an immortalized foreskin fibroblast cell line with or without oncogenic HRAS [3]. Similar effects are observed with retinal pigment epithelial cells, hepatocellular and colorectal carcinoma upon treatment with BSO [11,12,13]. However, this connection between GSH and ferroptosis is also frequently an indirect conclusion drawn from the fact that both xCT- and GPx4- inhibition result in ferroptosis, which can be quite misleading. Namely, a recent report of Harris and colleagues [14], in which the authors examined data from genome-scale pooled CRISPR-Cas9 screens of more than 300 cell lines, showed that actually only a very small subset of cell lines were sensitive to the inhibition of GSH biosynthesis, which does not seem to correlate with the sensitivity of these cells to xCT or GPx4 inhibition. Therefore, in order to identify the best potential target for ferroptosis induction, it is of the utmost importance to understand the role and the significance of individual players in this type of cell death.

Previous data from our own and other teams unambiguously showed the high sensitivity of the pancreatic ductal adenocarcinoma (PDAC) to ferroptosis induced by cysteine starvation or by pharmacological/genetic inhibition of xCT transporter [15,16,17]. Each of these interventions seems to be linked with a rapid decline in the intracellular GSH level. Nonetheless, to date, there is no study showing that GSH depletion alone could induce ferroptosis in PDAC cells. Considering the wide variety of roles played by cysteine [18], it is not completely clear which part of this sensitivity is GSH-dependent and which part is GSH-independent. To investigate this issue, we generated GCLc knockout cell lines starting from the previously used and described Capan-2 and MiaPaCa-2 cells [15]. Data obtained in the study showed that genetic deletion of the key enzyme in GSH biosynthesis induces a marked oxidative stress, suppressing proliferation and sensitizing PDAC cells to cell death. Nonetheless, the major pathway triggered by this genetic modification is apoptotic cell death, while the characteristic features of ferroptosis (lipid hydroperoxides, rescue with vitamin E) were absent. Furthermore, we showed that GPx4 enzyme remains active and functional in the GCLc-KO cells, most likely thanks to the reducing power of other free cellular thiols, in the absence of GSH. Finally, in vivo data revealed that targeting GSH biosynthesis might not have the desired impact on the tumor growth and survival, although the potential impact of GSH depletion could be visible if combined with chemotherapy. In conclusion, the data presented here suggest that the role GSH plays in ferroptosis is dispensable, but the impact of GSH-depletion might be a potent strategy for sensitizing cancer cells to the pro-oxidative chemotherapeutics.

## 2. Material & Methods

### 2.1. Cell Culture

Human Pancreatic Ductal Adenocarcinoma (PDAC) MiaPaCa-2 and Capan-2 cells were grown at 37 °C/5% CO_2_ in DMEM (Gibco) supplemented with 7.5% FBS (Dutscher Bernolsheim, France), penicillin (10 U/mL), and streptomycin (10 μg/mL) (Thermo Fisher Scientific, Waltham, MA, USA). Human PDAC MiaPaCa-2 and Capan-2 cells were kindly provided by Dr. Sophie Vasseur (CRCM, Marseille, France), who obtained them from ATCC, and authenticated in 2015. GCLc-KO cell lines in both Capan-2 and MiaPaCa-2 were maintained in DMEM media supplemented with freshly prepared 3 mM GSH in dH_2_0 (Reduced Glutathione, Sigma-Aldrich, St. Louis, CA, USA) and 100μM β-Mercaptoethanol (β-ME, Sigma-Aldrich, St. Louis, CA, USA). 

### 2.2. Genomic Disruption of GCLc and GPx4 Using CRISPR-Cas9 

MiaPaCa-2 and Capan-2 WT cells were transfected with PX458 plasmids containing CRISPR-Cas9. Targeting regions of the exons 1 and 3 of the GCLc, i.e., exons 3 and 4 of the GPx4, gene was conducted using Nucleofection^®^ (Lonza Cologne AG, Bâle, Switzerland). Guides used are presented in Table 1 at the end of Section 2. As the pSpCas9(BB)-2A-GFP (PX458) plasmid (plasmid #48138, Addgene, Watertown, MA, USA) used in the study contains GFP, single-cell sorting was conducted (24-h post transfection) using BD FACS Aria (BD Biosciences, Franklin Lakes, USA). Individual clones were cultivated in presence of 3 mM GSH + 100 μM β-ME (for GCLc), i.e., 10 μM vitamin E (for GPx4). Each individual clone was analyzed for GCLc, i.e., GPx4 expression by immunoblotting and ARD1 or tubulin were used as a loading control. 

### 2.3. GSH Measurement 

Cells (40,000 per well, duplicate per condition) were seeded in 96-well plates at 37 °C/5% CO_2_ in DMEM supplemented or not with different compounds (3 mM GSH, 10 mM GSH, 3 mM GSH + 100 μM β-ME, 3 mM GSH ethylester (GSHEE, Sigma-Aldrich, St. Louis, CA, USA), 1 mM N-acetylcysteine (NAC, Sigma-Aldrich, St. Louis, CA, USA), 150 μM gamma-glutamylcysteine (γ-GluCys, Sigma-Aldrich, St. Louis, CA, USA)). Intracellular GSH level was determined after 24 and 48 h by SAFAS Xenius XOF (Safas) using the GSH fluorimetric assay kit (CS1020, Sigma-Aldrich, St. Louis, CA, USA) according to the manufacturer’s instructions. Relative GSH content was normalized to the number of cells. 

### 2.4. Proliferation Assay

The different cell lines (2.5 × 10^4^ cells) were seeded in 6-well plates in triplicate per cell line and per condition. We measured proliferation by trypsinizing cells and counting them daily with a Coulter Z1 (Beckman, Brea, CA, USA) after 24 h, 48 h, 72 h, 96 h, and 120 h. The cell proliferation index was calculated as “fold of change” by standardizing each measurement to the cell number obtained 4 h post-seeding (corresponding to day 0). 

### 2.5. Clonogenicity Assay 

The different cell lines (1000 cells for Capan-2 and 3000 cells for MiaPaCa-2) were seeded in 60 mm dishes in control or supplemented condition and incubated at 37 °C, 5% CO_2_. After 10 or 17 days, dishes were stained with 5% Giemsa (Fluka) for 30–45 min to visualize colonies. 

### 2.6. Immunoblotting

Cells were lysed in 1.5 × Laemmli buffer, and protein concentrations were determined using the Pierce BCA protein assay (Thermo Scientific, Waltham, MA, USA). Hence, 40 μg protein extracts were separated by electrophoresis on 10% SDS polyacrylamide gel and transferred onto polyvinylidene difluoride membranes (Millipore, Burlington, MA, USA). Membranes were blocked in 5% non-fat milk in TN 1X buffer (50 mM Tris- HCl, pH 7.4, 150 mM NaCl) and incubated with the following anti-human antibodies: GCLc (1:1000, ab53179 Abcam, Cambridge, UK), GPx4 (1:1000, ab125066 Abcam, Cambridge, UK), xCT (1:1000, 12691S Cell Signaling, Danvers, MA, USA), NRF2 (1:1000, ab137550 Abcam, Cambridge, UK), p-GCN2 kinase (1:1000, ab75839 Abcam, Cambridge, UK), ATF4 (1:1000, 11815S Cell Signaling, Danvers, MA, USA). Homemade antibody against ARD1 [19] or tubulin (1:10,000 MA5-16308, Thermo Scientific, Waltham, MA, USA) were used as loading controls. Immunoreactive bands were detected with horseradish peroxidase anti-mouse or anti-rabbit antibodies (Promega) using the ECL system (Merck Millipore WBKLS0500, Burlington, MA, USA) and readings were performed using the LI-COR Odyssey Imaging System. 

### 2.7. Cell Death and Lipid Hydroperoxidation 

A BD FACS Melody cytometer was used. Experiments were performed at least three times, 10 000 events were collected per sample and analyzed by the FlowJo software package. For data presentation the modal scaling option was used (each peak is normalized to its mode, i.e., to% of maximal number of cells found in a particular bin). 

Cell death: Cells were collected by trypsinization, merged with the corresponding supernatant, and collected by centrifugation. Cell pellets were suspended in FACS buffer (PBS, 0.2% BSA, 2 mM EDTA) and stained with 2 μg/mL propidium iodide (Invitrogen, Waltham, MA, USA). PI was added 1 min before analysis.

Detection of lipid hydroperoxides: BODIPY^TM^ 581/591 C11 (Molecular Probes, Eugene, OR, USA) dye was added in the media to the final concentration of 2μM and the cells were incubated for 30 min at 37 °C/5% CO_2_ protected from the light. Subsequently, cells were washed two times with PBS, detached using accutase (Dutscher, Bernolsheim, France), and resuspended in FACS buffer. 

Different cell lines were seeded in 6-well plates (50,000 cells for Capan-2 and 100,000 cells for MiaPaCa-2 per well, triplicate per condition) in DMEM supplemented or not with 3 mM GSH + 100 μM β-ME, 10 μM vitamin E (α-Tocopherol, T1539 Sigma-Aldrich, St. Louis, CA, USA), 1 mM NAC, 20 μM Q-VD-Oph (258024, Selleckchem, Radnor, PA, USA), and/or 0.2 μM RSL3 (SML2234, Sigma-Aldrich, St. Louis, CA, USA). 

### 2.8. ROS Analysis 

ROS level was measured by staining with dichlorofluorescine diacetate (DCFDA) following commercial kit instructions (Cellular ROS Assay kit, ab113851 Abcam, Cambridge, UK) and analyzed by BD FACS Melody after 24, 48, or 72 h. 

### 2.9. Tumor Xenograft Studies 

Animal housing was conducted in compliance with the EU directive 2010/63/EU. Briefly, each cage contained 5 mice with an enriched environment. Food and water were given *ad libitum*, and the litter was changed on a weekly basis. Animal care met the EU directive 2010/63/EU ethical criteria. The animal experimental protocol was approved by the local animal care committee (Veterinary Service and Direction of Sanitary and Social Action of Monaco; Dr. H. Raps, Centre Scientifique de Monaco, Monaco). Capan-2, WT, and GCLc-KO stable cell lines (1 × 10^6^ cells) were suspended in 200 μL of High protein concentration Matrigel (CorningTM, Thermo Fisher, Waltham, MA, USA) and serum-free DMEM (final protein concentration 8 mg/mL) supplemented with insulin–transferrin–selenium (Life Technologies, Carlsbad, CA, USA). In both WT and GCLc-KO, cell preparations were supplemented with 3 mM GSH before cells were injected subcutaneously into the back of 8-week-old female athymic mice (Janvier) on the left and right side. Tumor dimensions were measured 2–3 times a week using calipers, and the tumor volume was determined using the formula: (4π/3) × L/2 × W/2 × H/2 (L, length; W, width; and H, height). When the tumor volume reached 1000–1500 mm^3^, mice were euthanized. At the end of the experiment, tumors from both WT and GCLc-KO groups were taken for protein extraction and Western blot analysis. 

### 2.10. Boyden Chamber

The LS174T WT cells were pre-seeded (5000 cells/well) in the upper well of the Boyden chamber (Falcon^®^ Permeable Support for 24-well Plate with 8.0 μm Transparent PET Membrane), and 24 h later, WT or GCLc-KO cells (MiaPaCa-2 or Capan-2) were added in the lower well, 30,000 cells/well for MiaPaCa-2 and 15,000 cells/well for Capan-2. After 96 h, the upper well was removed and the guest cells from the lower well were analyzed for cell death (BD FACS Melody analysis).

### 2.11. Statistical Analysis

Data are expressed as mean ± SEM. Each experiment was performed at least three times. Statistical analysis was performed with the unpaired Student *t* test. Differences between groups were considered statistically significant when *p* < 0.05.

## 3. Results

### 3.1. Genetic Deletion of the Catalytic Subunit of GCL Leads to the Collapse of GSH Intracellular Pool and Abolishes Growth of the PDAC Cell Lines

**Figure 1:**
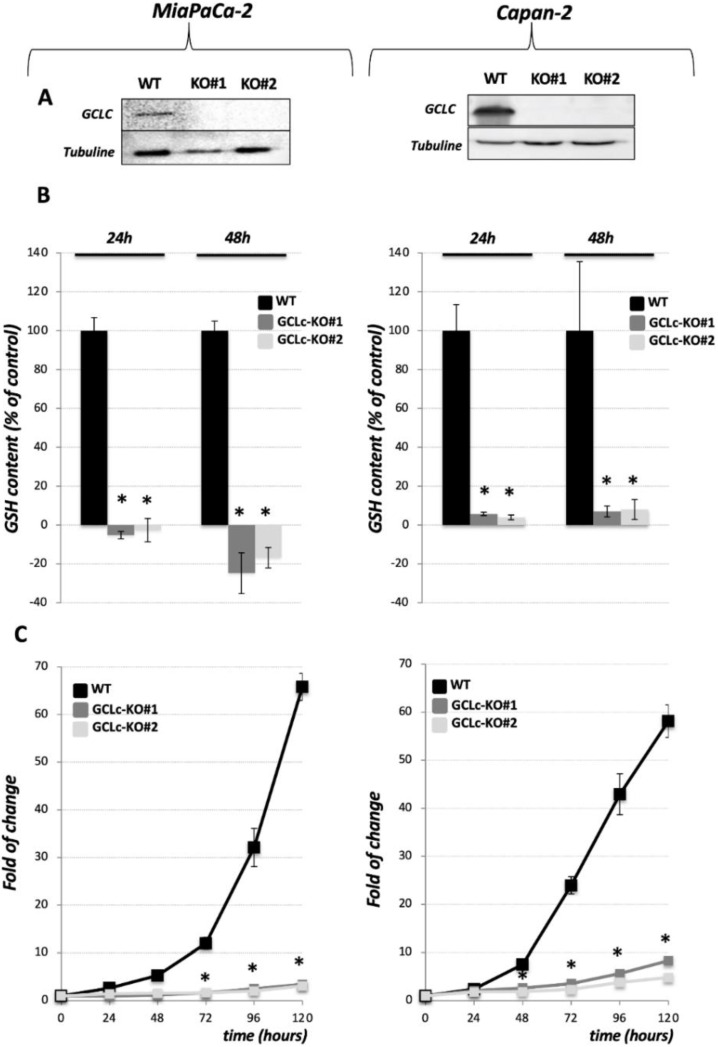
Gamma-glutamylcysteine (GCL) ligase determines cellular GSH content and cell proliferation of PDAC cells. (**A**). The expression of catalytic subunit of GCL (GCLc) was analyzed in MiaPaCa-2 and Capan-2 WT and two independent GCLc-KO cell lines (GCLc-KO#1 and GCLc-KO#2). Three independent experiments were performed, and representative blots are shown. (**B**). Relative intracellular GSH level was measured in WT and GCLc-KO cells of two PDAC cell lines after 24 h and 48 h upon seeding in the standard DMEM media. GSH content was normalized to the number of cells. Data shown represent the mean ± SEM; *n* = 3, * *p* < 0.05, comparison with corresponding WT control. (**C**). Proliferation of MiaPaCa-2 and Capan-2 WT and GCLc-KO cells seeded in the standard DMEM conditions. Proliferation rates are presented as fold of change (mean ± SEM; *n* = 3), * *p* < 0.05, comparison with WT control cells.

The catalytic subunit of the GCL (GCLc) was genetically deleted in two PDAC cell lines: MiaPaCa-2 (Figure 1, left side of the figure) and Capan-2 (Figure 1, right side of the figure), using CRISPR-Cas9. Analysis of genomic DNA and sequencing of the CRISPR-targeted site demonstrated disruptive mutations in the GCLc that caused the lack of corresponding protein expression in GCLc-KO clones of both cell lines, as shown by Western blot analysis (Figure 1A and Figure S5). Clones were grown in the presence of 3 mM GSH and 100 μM β-ME. An interesting change in the morphology of Capan-2 GCLc-KO cells was observed (Supplementary Figure S1). Namely, Capan-2 WT cells have an epithelial appearance characterized by the high expression of E-cadherin [15], while the morphology of derived GCLc-KO cells was shifted toward a more mesenchymal phenotype, as seen on the micrographs depicted in Supplementary Figure S1 (white arrows). On the other side, the MiaPaCa-2 cell line is more mesenchymal and no change in the appearance of its GCLc-KO cells was observed. 

Considering the pivotal role that GCLc plays in the synthesis of GSH, we investigated the content of this highly abundant antioxidant in the GCLc-KO and their parental cell lines. GCLc-KO cells of both cell lines showed a complete collapse of intracellular GSH at 24 h and 48 h after seeding in the absence of external GSH (Figure 1B and Figure S5). Interestingly, the content of the GSH had not been replenished in these cell lines even if the media was supplemented with exogenous GSH, GSH + 100 μM β-ME, a more efficiently transported form of GSH—GSH ethyl ester (GSHEE), N-acetylcysteine (NAC), or the product of GCL enzyme, dipeptide γ-glutamyl-cysteine (γ-GluCys) (Supplementary Figure S2A,B).

In both PDAC cell lines, the GCLc deletion significantly reduced the proliferation and clonogenicity potential of the cells growing in the standard DMEM media (Figure 1C, Supplementary Figure S3A,B). More precisely, the proliferation of GCLc-KO cell lines was completely suppressed, when the cells were cultivated in the control conditions for five days (Figure 1C, gray lines). Moreover, no visible clones were detected after 10 and 17 days in clonogenicity assays of GCLc-KO cells (Supplementary Figure S3A, first row). On the other side, if the media was supplemented with 3 mM GSH, the proliferation of the GCLc-KO cells was substantially increased in the proliferation assay (96 h, Supplementary Figure S3B), but not in the clonogenicity assay (10 and 17 days, Supplementary Figure S3A), although a few small clones Capan-2 GCLc-KO appeared after 17 days in these conditions. The addition of GSH and reducing agent, β-ME, had a similar effect when proliferation was followed for 96 h (Supplementary Figure S2B). However, the combination of GSH and β-ME allowed GCLc-KO to form more numerous and bigger colonies after 10 and 17 days (Supplementary Figure S3A). Nonetheless, the proliferation and clonogenicity potential of GCLc-KO cells in either cell line was never restored to control level. 

### 3.2. Genetic Deletion of GCLc Leads to Non-Ferroptotic Cell Death and Is Not Characterized by Accumulation of Lipid Hydroperoxides 

**Figure 2:**
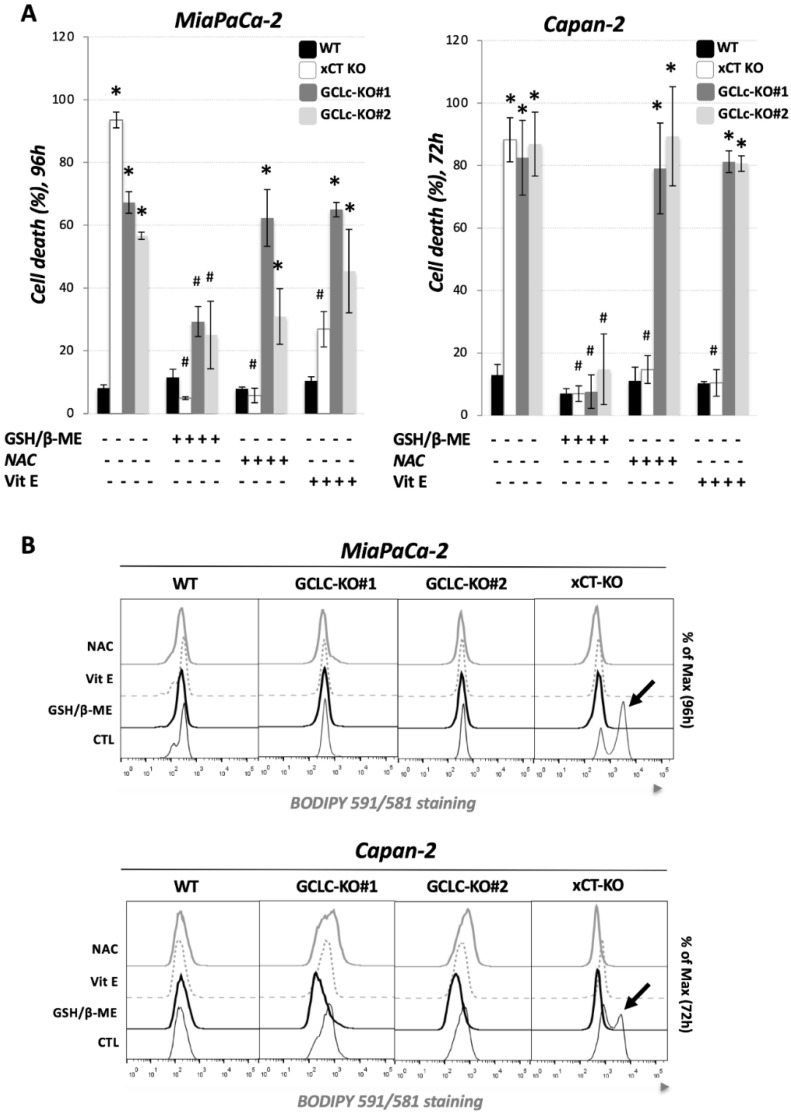
Genetic deletion of GCLc does not induce ferroptosis in PDAC cell lines. (**A**). Cell viability was measured using PI staining method (see Material & Methods). Cells were seeded in media supplemented or not with 3 mM glutathione (GSH) + 100 μM β-mercaptoethanol (β-ME), 1 mM N-acetylcysteine (NAC) or 10 μM vitamin E (Vit E), and cell viability was analyzed after 72 h (for Capan-2), i.e., 96 h (for MiaPaCa-2). The results are presented as mean ± SEM, *n* = 3. * *p* < 0.05, comparison with WT cells in control conditions; ^#^ *p* < 0.05, comparison with corresponding cell line in control conditions. (**B**). Lipid hydroperoxide content was measured in WT, GCLc-KO and xCT-KO cells 72 h (for Capan-2), i.e., 96 h (for MiaPaCa-2) upon seeding in media supplemented or not with 3 mM glutathione (GSH) + 100 μM β-mercaptoethanol (β-ME), 1 mM N-acetylcysteine (NAC) or 10 μM vitamin E (Vit E). xCT KO cells were used as ferroptotic positive control (black arrow) based on our previously published data (see the text). Presented histograms are representative of three independent experiments.

As the disruption of GCLc completely abolished the proliferation and clonogenicity potential of GCLc-KO cells, and considering the well-known key role of GSH in ferroptotic cell death, we further investigated if GCLc disruption leads to cell death and if so, whether it could be prevented by known inhibitors of ferroptosis. We used the previously described xCT-KO cell line [15] as a positive control for ferroptotic cell death. Observation of MiaPaCa-2 and Capan-2 GCLc-KO cells seeded in the DMEM media revealed an approximately 60% and 80% reduction in viability after 96 h and 72 h, respectively (Figure 2A, grey bars). The combination of GSH and β-ME completely prevented cell death in both cell lines, while the addition of an alternative source of cysteine (NAC) or lipophilic antioxidant (vitamin E) had no effect on cell death in GCLc-KO. A noticeable difference between xCT-KO (Figure 2A, white bars) and GCLc-KO (Figure 2A, gray bars) cells, of the same cell line, was observed both at the level of cell death kinetic, as well as regarding the rescue effects of NAC and Vitamin E, which completely prevented the cell death of xCT-KO cells. 

One of the major molecular markers of ferroptosis is the accumulation of oxidative damage in the membrane lipids. We investigated the level of lipid hydroperoxides in WT, GCLc-KO, and xCT-KO (positive control) cell lines and observed no significant increase in the lipid hydroperoxides content in GCLc-KO cells of both cell lines after 72 or 96 h (or earlier; data not shown), at the point of a massive cell death (Figure 2B), which was a strikingly different profile to the one observed in with xCT-KO cells. Considering no increase in BODIPY 591/581 C11 signal in control conditions of GCLc-KO cells, it came as no surprise that NAC and Vitamin E had no impact on the lipid hydroperoxide content of these cells, while in the xCT-KO cells they both completely restored not only the lipid hydroperoxides content (Figure 2B), but also viability, as mentioned previously (Figure 2A). These data collectively suggested that GCLc-KO cells, although vulnerable, resist the uncontrolled lipid hydroperoxide accumulation and ferroptotic cell death, which will be investigated in more detail later.

### 3.3. Glutathione Depletion Sensitizes Cells to ROS-Dependent Apoptosis but Not to Ferroptosis Cell Death

**Figure 3:**
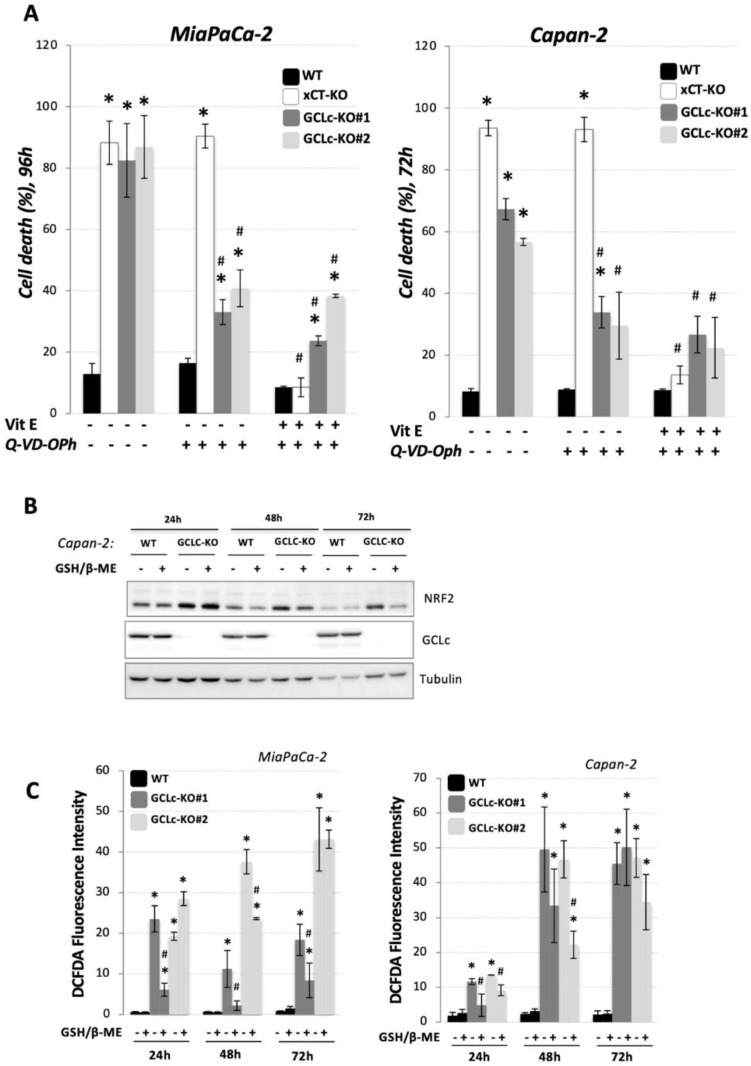
Glutathione depletion *per se*, leads preferentially to apoptosis in PDAC cells. (**A**). Cell viability was measured using PI staining method (see Material & Methods). Cells were seeded in media supplemented or not with ferroptosis inhibitor (10 μM Vit E), apoptosis inhibitor (20 μM Q-VD-Oph) or their combination, and cell viability was analyzed after 72 h (for Capan-2), i.e., 96 h (for MiaPaCa-2). xCT-KO cells were used as ferroptotic positive control (*white bars*) based on our previously published data (see the text). These results present mean ± SEM, *n* = 3. * *p* < 0.05, comparison with WT cells in control conditions; ^#^ *p* < 0.05, comparison with corresponding cell line in control conditions. (**B**). Protein content of a major sensor of oxidative stress (nuclear factor (erythroid-derived 2)-like 2 or NRF2) was analyzed in Capan-2 WT and GCLc-KO cells seeded in standard DMEM media supplemented or not with 3 mM GSH + 100 μM β-ME, after 24, 48 and 72 h. Blots are representative of three independent experiments. (**C**). Intracellular ROS level was measured by staining with DCFDA (see Material & Methods) in WT and GCLc-KO cells of two PDAC cell line 24, 48 and 72 h after seeding in media supplemented or not with 3 mM GSH + 100 μM β-ME. The results are presented as mean ± SEM, *n* = 3. * *p* < 0.05, comparison with corresponding WT control; ^#^ *p* < 0.05, comparison with corresponding cell line in control conditions.

GSH is the most abundant non-enzymatic antioxidant in the cell, and as such, its depletion has been linked to many different types of cell death, including apoptosis [20]. In order to investigate whether the disruption of GCLc in two PDAC cell lines leads to apoptotic cell death, the cells were treated with the well-known and potent pan-caspase inhibitor, Q-VD-Oph (Figure 3A), following cell death measurement after 72 h for Capan-2, i.e., 96 h for MiaPaCa-2 (time points previously determined (Figure 2A)). Q-VD-Oph decreased cell death from 60–80% to approximately 30–40% in both PDAC GCLc-KO cell lines, suggesting that the majority of the cell viability loss of GCLc-KO cells is due to apoptosis. On the contrary, Q-VD-Oph had zero effect on the cell death of xCT-KO cells, coming from the same parental cell lines. The combination of apoptosis and a ferroptosis inhibitor had no additional rescue effect in comparison with Q-VD-Oph (for GCLc-KO) or Vitamin E (for xCT-KO) alone, suggesting completely different cell death pathways in these two genetically modified cell lines.

Next, we analyzed the effects of GSH depletion by the genetic disruption of GCLc on the redox status of PDAC cells. Firstly, we analyzed the protein content of the major redox-sensitive transcriptional factor, nuclear factor (erythroid-derived 2)-like 2 (NRF2), after 24, 48, and 72 h, in the presence or not of exogenous GSH (3 mM) + β-ME (100 µM). As depicted in Figure 3B, NRF2 was detected significantly above the control level in GCLc-KO cells at all investigated time points (24, 48, and 72 h) in the absence of exogenous GSH in media (Figure 3B), while the addition of GSH + β-ME slightly, but not completely, restituted the level of NRF2 in these cells. Furthermore, FACS analysis, upon DCFDA staining, showed that GCLc-KO cells of either cell line have a significantly higher ROS level (Figure 3C, gray bars) when compared to the parental (WT) cell lines (Figure 3C, black bars). Interestingly, the addition of exogenous GSH + β-ME did not uniformly and consistently prevent ROS accumulation in GCLc-KO cells, especially after a prolonged period in the culture (72 h). 

### 3.4. GCLc-KO Cells Remains Highly Sensitive to GPx4 Inhibition 

**Figure 4:**
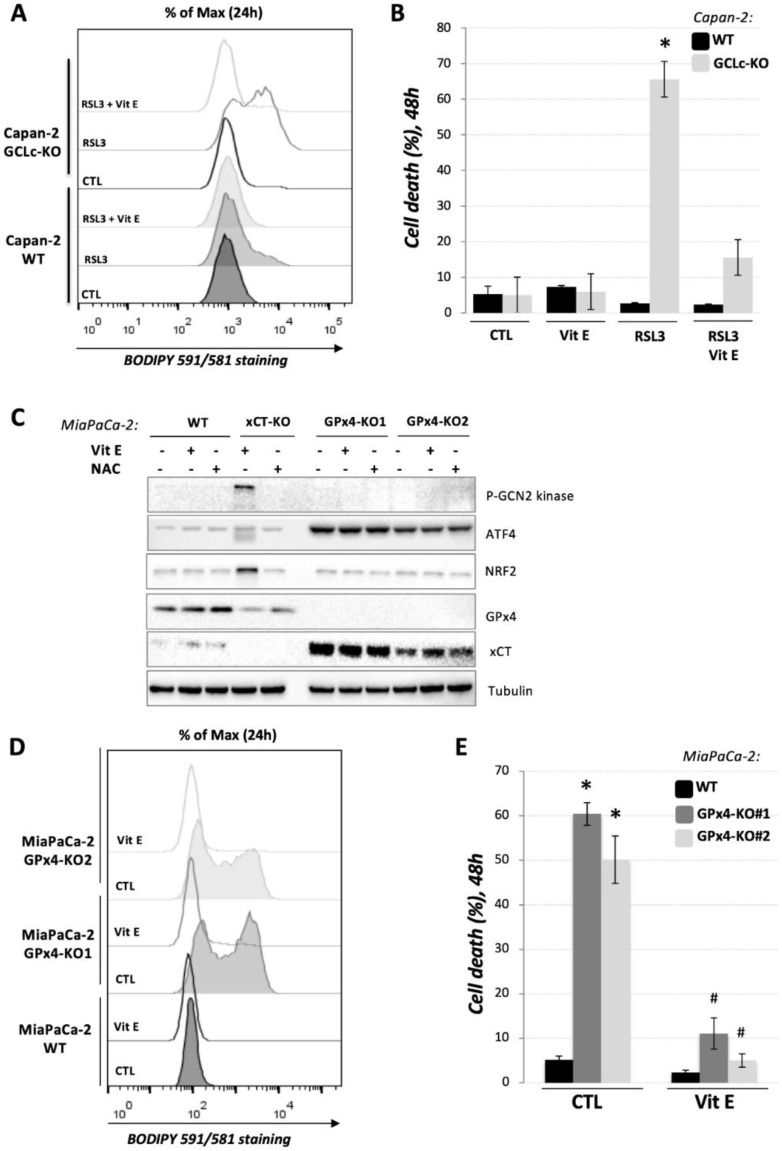
GPx4 enzyme is functional in GCLc-KO cells of both PDAC cell lines. Capan-2 WT and GCLc-KO cells were treated with 0.2 μM inhibitor of GPx4 enzyme (RSL3) and (**A**). lipid hydroperoxide content and (**B**). cell viability was analyzed after 24 h, i.e., 48 h, respectively. Combination of RSL3 (0.2 μM) and vitamin E (10 μM) was used as a control for specificity of RSL3 effects. The results are presented as mean ± SEM, *n* = 3. * *p* < 0.05, comparison with corresponding WT control. Presented histograms are representative of three independent experiments. (**C**). MiaPaCa-2 WT, xCT-KO and GPx4-KO cells were cultivated for 24 h in DMEM supplemented or not with 10 μM vitamin E or 1 mM NAC. Changes in phosphorylation status and protein abundance of the major sensors of AA-depletion (p-GCN2 and ATF4) and/or oxidative stress (NRF2 and ATF4), as well as two major players in ferroptotic cell death (xCT and GPx4) were analyzed by Western blot. Blots are representative of three independent experiments. (**D**). Lipid hydroperoxide content and (**E**) cell viability was analyzed after 24 h, i.e., 48 h, respectively, in MiaPaCa-2 WT and GPx4-KO cell lines seeded in media supplemented or not with 10μM vitamin E. * *p* < 0.05, comparison with corresponding WT control; ^#^ *p* < 0.05, comparison with corresponding cell line in control conditions.

Given that GSH serves as a reducing power for the GPx4 enzyme to neutralize the oxidative damage of lipids in the plasma membrane and thus prevent ferroptosis [3,21], one of the major questions concerned how GCLc-KO cells, in the absence of GSH, prevent the accumulation of lipid hydroperoxides. Considering the recent identification of FSP1/ubiquinol and BH4/dihydrofolate reductase systems as alternative pathways for the detoxification of lipid hydroperoxides [22,23,24], one possible explanation was that GPx4 is actually dispensable for PDAC cells. To investigate this issue, we treated Capan-2 GCLc-KO cells with the potent GPx4 inhibitor RSL3 [25] and analyzed the lipid hydroperoxide content (Figure 4A) and cell death (Figure 4B) after 24 h, i.e., 48 h, respectively. Data obtained by FACS analysis upon BODIPY 581/591 C11 and PI staining showed that GCLc-KO cells are highly sensitive to GPx4 inhibition. Namely, RSL3-treatment induced a similar ferroptotic profile in GCLc-KO cells as the previously reported genetic and pharmacological inhibition of xCT transporter [15], while at the same time, the concentration of RSL3 (0.2 μM) had no effect on the parental Capan-2 cell line (Figure 4A,B). 

To further investigate this issue, we generated MiaPaCa-2 GPx4-KO cells that were in the presence of vitamin E in order to support cell survival (Figure 4C). Western blot analysis showed that these cells, oppositely to xCT-KO cells, do not exhibit classical amino acid or oxidative stress, based on the unchanged level of the major regulators involved in the response to these conditions, phospho-GCN2 (general control nonderepressible 2) kinase and NRF2. Nonetheless, the activation of ATF4 transcriptional factor in the absence of activated GCN2 kinase suggests a redox imbalance in the GPx4-KO cells and is most likely responsible for a strong up-regulation of xCT expression observed in the GPx4-KO clones (Figure 4C) [26,27]. The proliferation rate and clonogenicity potential of GPx4-KO cells were significantly reduced (Supplementary Figure S4A,B, respectively). The addition of vitamin E partially restituted both parameters, while β-ME allowed the formation of very small GPx4-KO clones. NAC and GSH, usually seen as alternative donors of cysteine, had profoundly different effects on clonogenicity potential of xCT-KO and GPx4-KO cells (Figure S4B).

**Figure 5:**
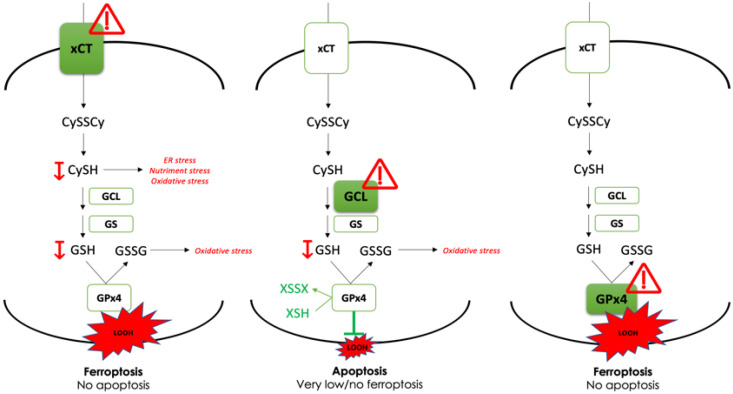
Proposed model of the study. Dissection of the main anti-ferroptotic axis (xCT-GSH-GPx4) in PDAC cell lines, using genetic tool (CRISPR-Cas9,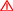), revealed indispensable role of cystine transporter (xCT) and antioxidant enzyme GPx4 for ferroptosis prevention. On the contrary, genetic deletion of the main enzyme in GSH biosynthesis (GCL) proved to be substitutable in this context (see Discussion).

Similar to RSL3 treatment of GCLc-KO, culturing the GPx4-KO cells for 24 h in standard DMEM media (control conditions) induced a strong accumulation of lipid hydroperoxides in GPx4-KO cell line (Figure 4D, grey lines), leading to the ferroptotic cell death of 50–60% of these cells after 48 h of culturing (Figure 4E, grey bars). Both effects were completely reverted by the addition of vitamin E in the media. Together, those results clearly showed a GSH-independent activity of GPx4 enzyme in the GCLc-KO cells (Figure 5). 

### 3.5. GCLc Is Dispensable for PDAC Cell Growth In Vivo 

**Figure 6:**
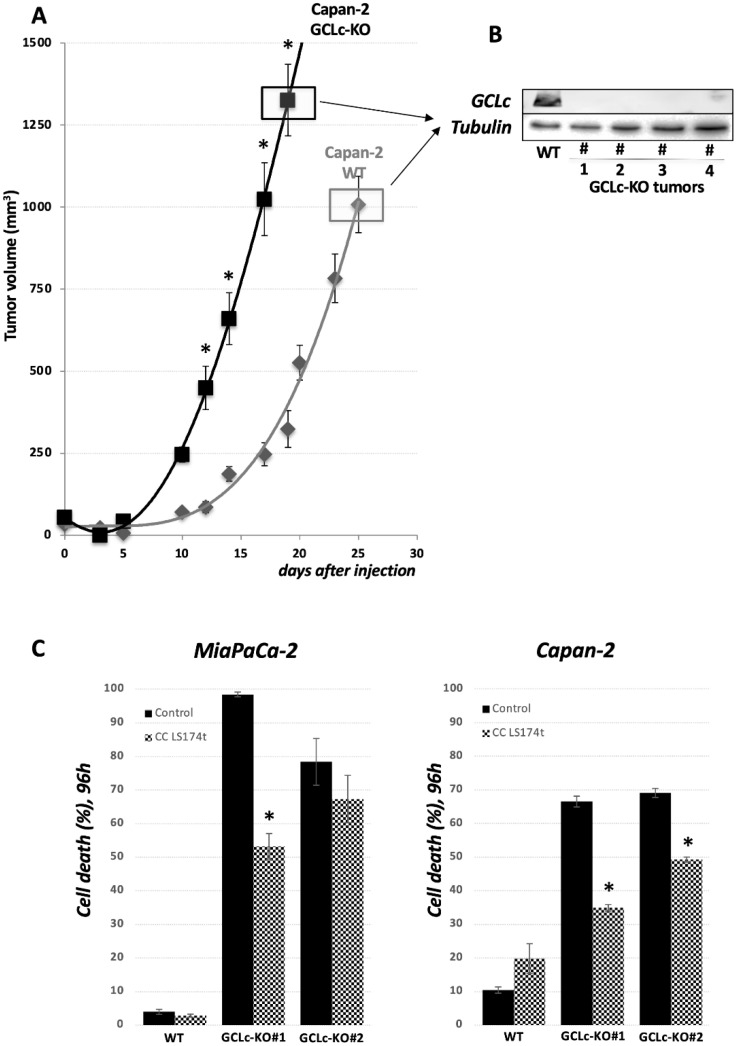
GCL is dispensable for tumor growth of PDAC cell lines in mice. (**A**). Tumor volumes of Capan-2 WT and GCLc-KO cells, injected subcutaneously into nude mice, showed slight increase of tumor growth upon GCLc genetic deletion. (**B**). At the end of the experiment, tumors are examined for the presence of GCLc protein. No GCLc protein band was detected in tumors isolated from Capan-2 GCLc-KO group at the end of the experiment. (**C**). Cell viability of MiaPaCa-2 and Capan-2 WT and GCLc-KO, co-cultured or not with 10% of LS174T WT cells (Boyden chamber, see Material & Methods), was analyzed after 96 h. The results are presented as mean ± SEM, *n* = 3. * *p* < 0.05, comparison with corresponding cell line in control conditions.

To investigate the importance of GCL enzyme and the GSH level to tumor growth in vivo, we performed xenograft experiments in nude mice. WT and GCLc-KO cells of Capan-2 cell lines treated with 3 mM GSH were injected subcutaneously with matrigel into nude mice and tumor growth was monitored. Surprisingly, as presented in Figure 6A, GCLc-KO cells are fully capable of forming tumors in vivo. Tumors isolated at the end of experiments were tested for the presence of GCLc enzyme, and no visible GCLc enzyme was observed in the tumors of GCLc-KO cells (Figure 6B). Based on our previous experience with xCT-KO cell growth in vivo, a potential explanation for GCLc-KO tumor cell survival and proliferation in the same conditions could be attributed to GSH exchange between GCLc-KO and the neighboring WT cells in the tumor mass. To investigate this issue, we used co-culture (cc) of Capan-2 or MiaPaCa-2 GCLc-KO cells and LS174T WT cells (cell line chosen based on the results from our previous study [28]). LS174T cells were pre-seeded in the upper well of a Boyden chamber 24 h before GCLc-KO cells, and the viability of the latter was investigated 96 h later (Figure 6C). According to the data, the survival of GCLc-KO cells seems to be partially reverted by the co-culture conditions, suggesting that cell-to-cell interplay may, at least partially, play a role in GCLc-KO survival in mice (Figure 6A). 

## 4. Discussion

The conceptualization of a completely novel, regulated type of cell death called ferroptosis opened a new avenue for the development of effective anti-cancer therapeutics [2]. Nonetheless, for tumor-selective ferroptosis inducers to be introduced in the clinic, full clarification of the major players and their role in ferroptosis represents the most important requirement. The key players, described up to now, include: (1) cystine-glutamate exchanger (x_c_- system); or more precisely its transport subunit, xCT, which is responsible for the import of the proteinogenic and redox-active amino acid, cysteine; (2) GSH, a nonenzymatic antioxidant, whose content is directly determined by the intracellular cysteine pool, and finally, (3) GPx4 enzyme, which uses GSH as a reducing power to detoxify lipid hydroperoxides, and thus directly prevent ferroptosis. Indeed, an overwhelming amount of literature data showed the great sensitivity of cancer cells across a wide range of tumor types to ferroptosis induction by xCT or GPx4 inhibition. However, the importance of GSH as a driving reducing power does not seem to be that conclusive. Namely, a recent report of Harris and colleagues revealed that only a very small subset of cell lines shows sensitivity towards pharmacological GSH depletion [14]. The authors argue that the resistance towards GSH depletion is linked with the deubiquitinating system of the cells that ensure protein homeostasis and cell viability. Nonetheless, the question that remained to be answered is what mechanism(s) allows the resistance of these cells to ferroptosis, i.e., do these cells prevent lipid peroxidation of the membrane lipids in the absence of GSH, and if so, how?

Results from our own and other laboratories unambiguously showed that cysteine depletion, either by cysteine withdrawal or by pharmacological/genetic inhibition of its major transport system (xCT) inevitably leads to GSH depletion, the accumulation of lipid hydroperoxides, and finally, ferroptosis in PDAC cell lines [15,16,17]. However, considering that cysteine, besides being the rate-limiting amino acid for GSH biosynthesis, plays a wide range of functions in the cells [4], it remains unclear if the ferroptotic phenotype associate with the cysteine-starvation is a simple result of GSH-depletion or not. To investigate this issue, we genetically deleted the catalytic subunit of the key enzyme involved in the GSH biosynthesis (GCLc) in two previously used and described PDAC cell lines [15] (Figure 1A). Similar to xCT-KO cells [15], GCLc-KO cells of both investigated cell lines were characterized by a complete collapse of GSH (Figure 1B), proliferation (Figure 1C), and survival (Figure 2A). However, opposite to xCT-KO, GCLc-KO cells showed two distinctive features: (1) kinetic of cell death was significantly slower, and (2) no visible markers of the ferroptotic cell death were observed. Namely, as previously reported, xCT-KO cells died by ferroptosis within 24/48 h, while GCLc-KO seemed to be more resistant and the near complete collapse of cell survival was visible after 72 h (Capan-2), i.e., 96 h (MiaPaCa-2). Surprisingly, the major inhibitor of ferroptosis, vitamin E [29], did not show a rescue effect in either of the GCLc-genetically deleted cell lines (Figure 2A). In line with these findings are results showing no apparent increase in lipid hydroperoxide content in the GCLc-KO cells, despite observed GSH depletion (Figure 2B). Although surprising, considering the previously reported high sensitivity of these cell lines to ferroptosis, these results corroborate with the complex and multiple role GSH plays in cell survival (reviewed in [20]). Maybe one of the most studied roles of GSH in this context is its involvement in repressing apoptosis (for further reading refer to [30]). Indeed, when pan-caspase inhibitor (Q-VD-Oph) was used, cell death of GCLc-KO cells was largely prevented, while no effect was observed in the xCT-KO cells (Figure 3A). One of the possible triggers of apoptosis in GCLc-KO cells seems to be oxidative disbalance, as seen through the increased protein content of the major redox sensitive transcriptional factor NRF2 (Figure 3B) and DCFDA staining (Figure 3C). This is in great accordance with the literature data reporting GSH depletion as both a marker and an inducer of oxidative stress leading to apoptosis [31,32].

Although oxidative stress induced by GSH-depletion and resulting in apoptosis is not surprising, interesting data obtained in the study suggest that exogenous GSH seems not to be enough to maintain cellular homeostasis in the GCLc-KO cells. Namely, cell proliferation (Supplementary Figure S3), intracellular GSH pool (Supplementary Figure S2), and redox balance (Figure 3C) have not been fully restituted by cultivating GCLc-KO cells in media supplemented with GSH. Furthermore, our initial attempts to grow potential GCLc-KO cells in media supplemented with GSH alone were unsuccessful. On the contrary, the addition of β-mercaptoethanol as a reducing equivalent allowed us to culture GCLc-KO and finally restitute their survival (Figure 2A), and to a certain degree, proliferation (Supplementary Figure S3). Nonetheless, the internal reserve of GSH has not been restored regardless of whether GSH or GSH + β-mercaptoethanol was added to the culture media. Moreover, we also investigated the other form of GSH, which is more effectively transported, GSH-ethylester, as well as the product of GCLc enzyme, γ-glutamylcystine, but without success. These results argue in favor of the great instability of GSH in the extracellular space, which seems to be promptly degraded into individual amino acids thanks to the activity of gamma-glutamyl transpeptidase (GGT) [33]. This refers to a process in which GSH is extracellularly degraded to individual amino acids that are consequently imported into the cells, leading, at least in part, to GSH biosynthesis, known as the Meister cycle [34]. Although the importance of the Meister cycle for GSH biosynthesis and regeneration has been under certain level of scrutiny (reviewed in [35]), our results provide corroboration, at least in principle.

When it comes to GCLc-KO cell resistance to ferroptosis, the major question posed concerns how these cells in the absence of GSH protect themselves from the uncontrolled accumulation of lipid hydroperoxides, and thus ferroptosis. A potential explanation for this came from two studies published back-to-back in 2019 [22,23] in which an alternative pathway of lipid hydroperoxide removal was described. This pathway includes coenzyme Q (CoQ, ubiquinol), which serves as a reducing power for the conversion of lipid hydroperoxides into lipid alcohols, and its regenerating enzyme, initially known as AIFM2 and then renamed to ferroptosis suppressor protein 1 (FSP1). Furthermore, this pathway seems to divorce the well-established axis GSH-GPx4 from cysteine transport, but itself might still be largely dependent on the xCT activity and cysteine pool. Specifically, by tracing the metabolism of exogenous ^13^C-labeled cysteine, Badgley et al. [17] showed that cysteine plays fundamental role not only in the GSH biosynthesis, but also the synthesis of CoQ precursor, coenzyme A (CoA), through the pantothenate pathway. This could explain the sensitivity of the PDAC cells toward cysteine depletion, even if an alternative CoQ-FSP1 antioxidant branch is present in the cells. Furthermore, in the study of Soula and coworkers [24], the tetrehydribiopterin (BH4)/dihydrofolate reductase system has been identified as yet another layer of cellular protection against lipid hydroperoxidation upon GPx4 inhibition. To investigate this hypothesis, and whether GSH-GPx4 pathway is indeed dispensable for ferroptosis prevention in PDAC cells, we treated Capan-2 GCLc-KO cells with a potent inhibitor of GPx4 (RSL3) and showed that these cells maintained sensitivity to GPx4 inhibition (Figure 4A,B). Furthermore, we generated a MiaPaCa-2 GPx4-KO cell line and observed a high sensitivity of these cells to ferroptosis (Figure 4C–E and Figure S4), very similar to the one observed with xCT-KO cells of the same cell lines [15]. These results led us to reject the hypothesis that an alternative GPx4-independent pathway is responsible for ferroptosis prevention in GCLc-KO cells. Nonetheless, the question regarding the activity of the GPx4 in the absence of GSH remained open, although alternative reducing equivalents for the GPx4 activity have already been proposed in the literature. As an example, Ursini’s group reported that mitochondrion-associated cysteine-rich proteins can be used as an alternative substrate for the GPx4 enzyme, while the same could be achieved by free cysteine or small thiol compounds [36,37]. In line with this hypothesis is the fact that the addition of small thiol (β-ME) in culture media allowed us to grow and culture GCLc-KO cells.

When it comes to in vivo conditions, tumor xenografts of GCLc-KO cells surprisingly grew somewhat faster in comparison with their WT counterpart (Figure 6). This could be attributed to the lower oxidative pressure that cells experience in these conditions. However, another possibility that became apparent in our previous work with xCT-KO xenografts is cell-to-cell interplay which, in the case of xCT-KO cells, provides them with the source of reduced cysteine [28,38]. Although the degradation of GSH in vivo is just as intensive as in vitro [35,39,40,41], the continuous flux of GSH from adjacent cells might be a sufficient source of GSH for GCLc-KO cells to survive and thrive in vivo. Indeed, our data showed that the co-culturing of GCLc-KO cells in the presence of the WT secretome had a partial rescue effect even in in vitro conditions (Figure 6C).

In conclusion, data obtained in the present study clearly showed that the genetic deletion of GCLc results in GSH-depletion, as well as suppressed proliferation and viability via the apoptotic cell death of PDAC cells. However, in contrast to xCT and GPx4, GSH seems to be dispensable for ferroptosis prevention, most likely due to the action of other thiol-containing molecules that can serve as an alternative donor of reducing power for the special seleno peroxidase GPx4. To the best of our knowledge, this is the first time that the effects of cysteine and GSH on ferroptosis have been divorced, and results unequivocally argue in favor of xCT as an efficient and selective target for ferroptosis induction in the case of pancreatic cancer.

## 5. Conclusions

In conclusion, data obtained in the present study clearly showed that the genetic deletion of GCLc results in GSH-depletion, as well as suppressed proliferation and viability via the apoptotic cell death of PDAC cells. However, in contrast to xCT and GPx4, GSH seems to be dispensable for ferroptosis prevention, most likely due to the action of other thiol-containing molecules that can serve as an alternative donor of reducing power for the special seleno peroxidase GPx4 (Figure 5). To the best of our knowledge, this is the first time that the effects of cysteine and GSH on ferroptosis have been divorced, and results unequivocally argue in favor of xCT as an efficient and selective target for ferroptosis induction in the case of pancreatic cancer.

## Figures and Tables

**Table 1:** gRNA guides used in the study.

*CrGCLc_ex1*	GC CAT GCC GAC CAC GTG CGG
*CrGCLc_ex3*	**G**C AAC ATG CGA AAA CGC CGG A
*CrGPx4_ex3*	**G**C CCG AAC TGG TTA CAC GGG A
*CrGPx4_ex4*	**G**A GAG ATC AAA GAG TTC GCC G

Bold G in 5’ is added due to the transcription initiation requirement of a ‘G’ base for human U6 promoter.

## Data Availability

The data is available upon request from corresponding author.

## Supplementary Materials

The following supporting information can be downloaded at: https://www.mdpi.com/article/10.3390/cancers14133154/s1. Figure S1: Characteristic morphology MiaPaCa-2 and Capan-2 WT and GCLc-KO cells; Figure S2: GSH content in WT and GCLc-KO cells of two PDAC cell lines; Figure S3: Proliferation and clonogenicity potential of WT and GCLc-KO cells of two PDAC cell lines; Figure S4: Proliferation and clonogenicity potential of WT and GPx4-KO cells of two PDAC cell lines; Figure S5: Original uncropped Western blots of Figure 1A and B, Figure 3B, Figure 4C, and Figure 5B.
