# Supplementary material for: Genetic Disruption of the γ-Glutamylcysteine Ligase in PDAC Cells Induces Ferroptosis-Independent Cell Death In Vitro without Affecting In Vivo Tumor Growth
Source: Cancers (Basel). 2022 Jun 28;14(13):3154. doi: 10.3390/cancers14133154 (PMC9264981; doi:10.3390/cancers14133154)
Supplement: Supplementary file 1 [file cancers-14-03154-s001.zip › cancers-1671481-supplementary.pdf]

Supplementary Materials: Genetic Disruption of the γ-Glutamylcysteine Ligase in PDAC Cells Induces Ferroptosis-Independent Cell Death In Vitro without Affecting In Vivo Tumor Growth

Boutaina Daher, Willian Meira, Jerome Durivault, Celia Gotorbe, Jacques Pouyssegur and Milica Vucetic

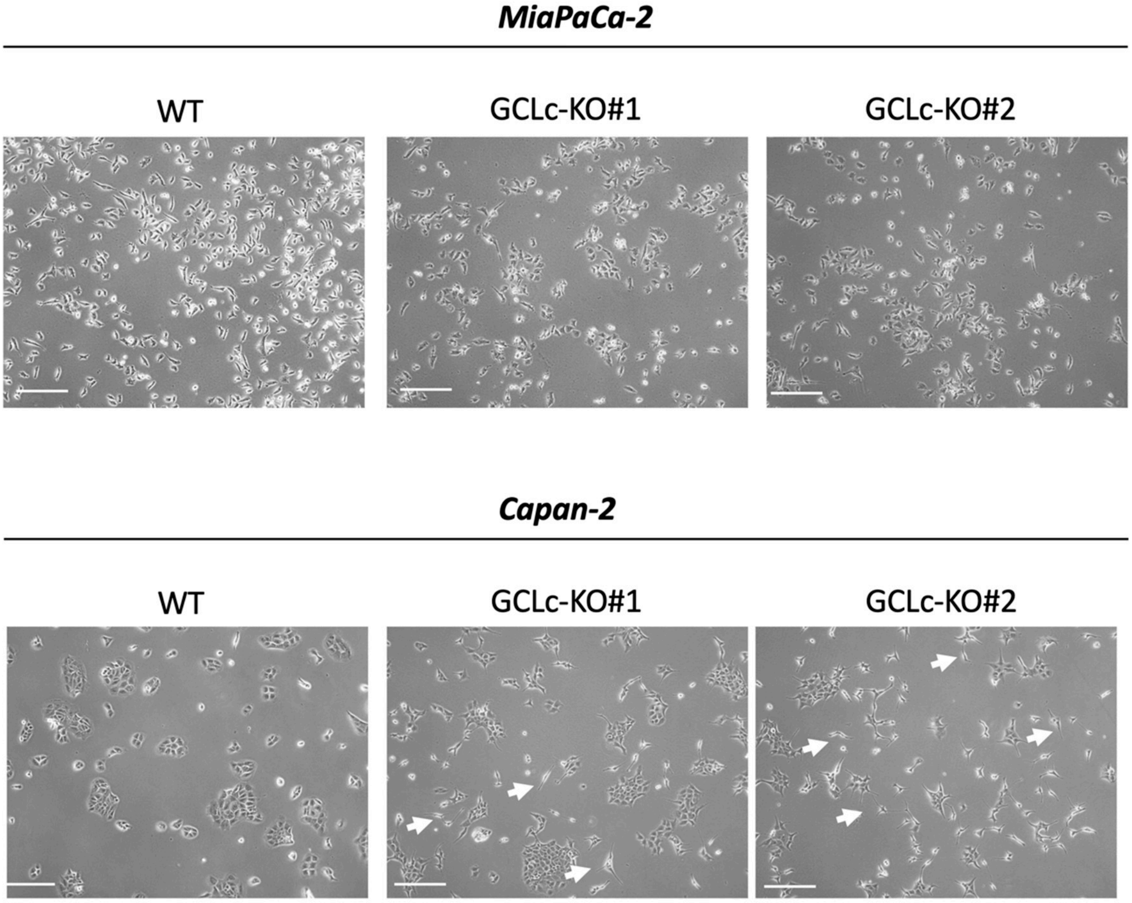

Figure S1. Characteristic morphology MiaPaCa-2 and Capan-2 WT and GCLc-KO cells. The clones cultured in the standard DMEM media supplemented with 3mM GSH + 100 M -ME. Mesenchymal features of the Capan-2 GCLc-KO are pointed with white arrows on the micrographs. Magnification 10x, scale bar – 50 m.

A

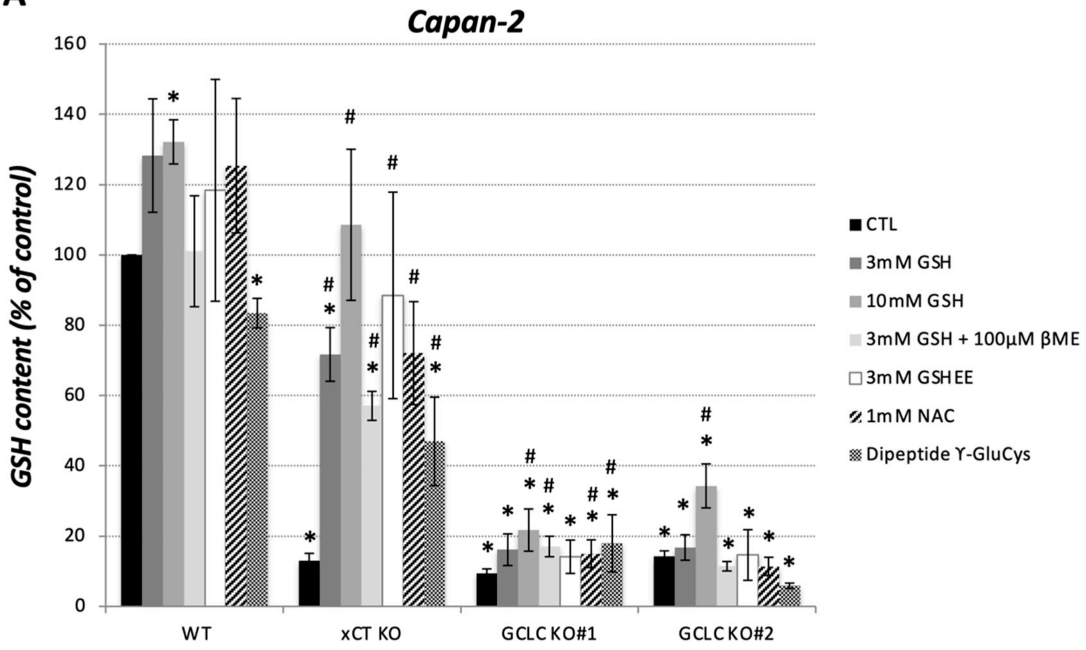

B

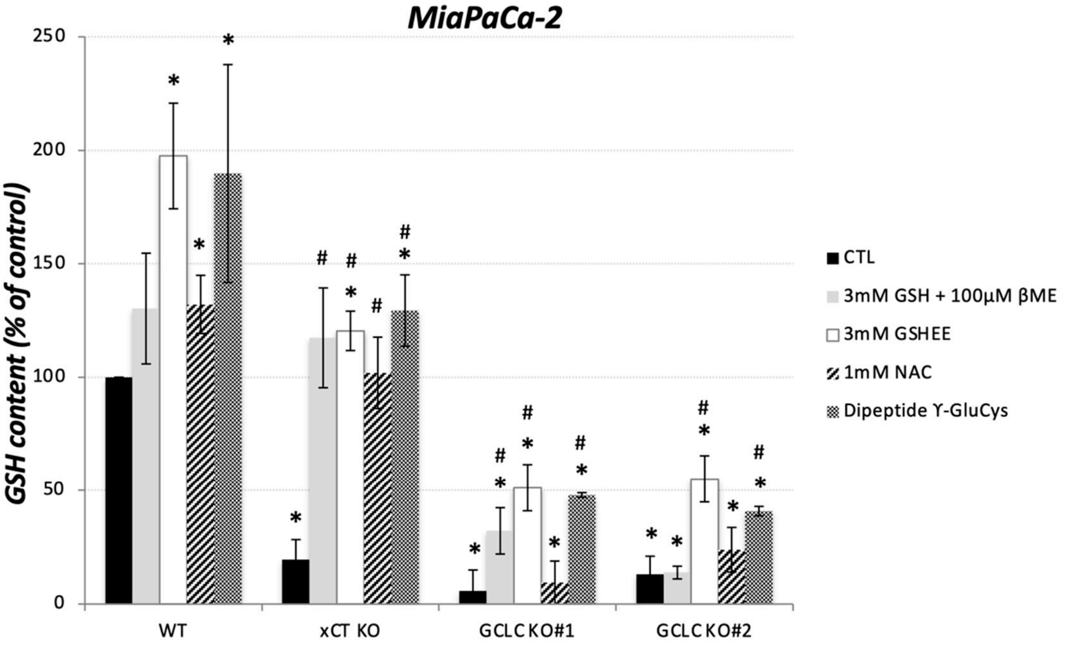

Figure S2. GSH content in WT and GCLc-KO cells of two PDAC cell lines. Relative intracellular GSH level was measured in Capan-2 (A) and MiaPaCa-2 (B) WT, xCT-KO and GCLc-KO cells 24h upon seeding in the standard DMEM media supplemented with 3mM or 10mM GSH, 3mM GSH + 100 M -ME, 3mM GSH ethylesyter (GSHEE), 1mM NAC or 200 M -glutamylcysteine (-GluCys). GSH content was normalized to the number of cells. Data shown represent the mean SEM; n=3, *P

< 0.05, comparison with corresponding WT control, #P < 0.05, comparison with corresponding cell line in control conditions.

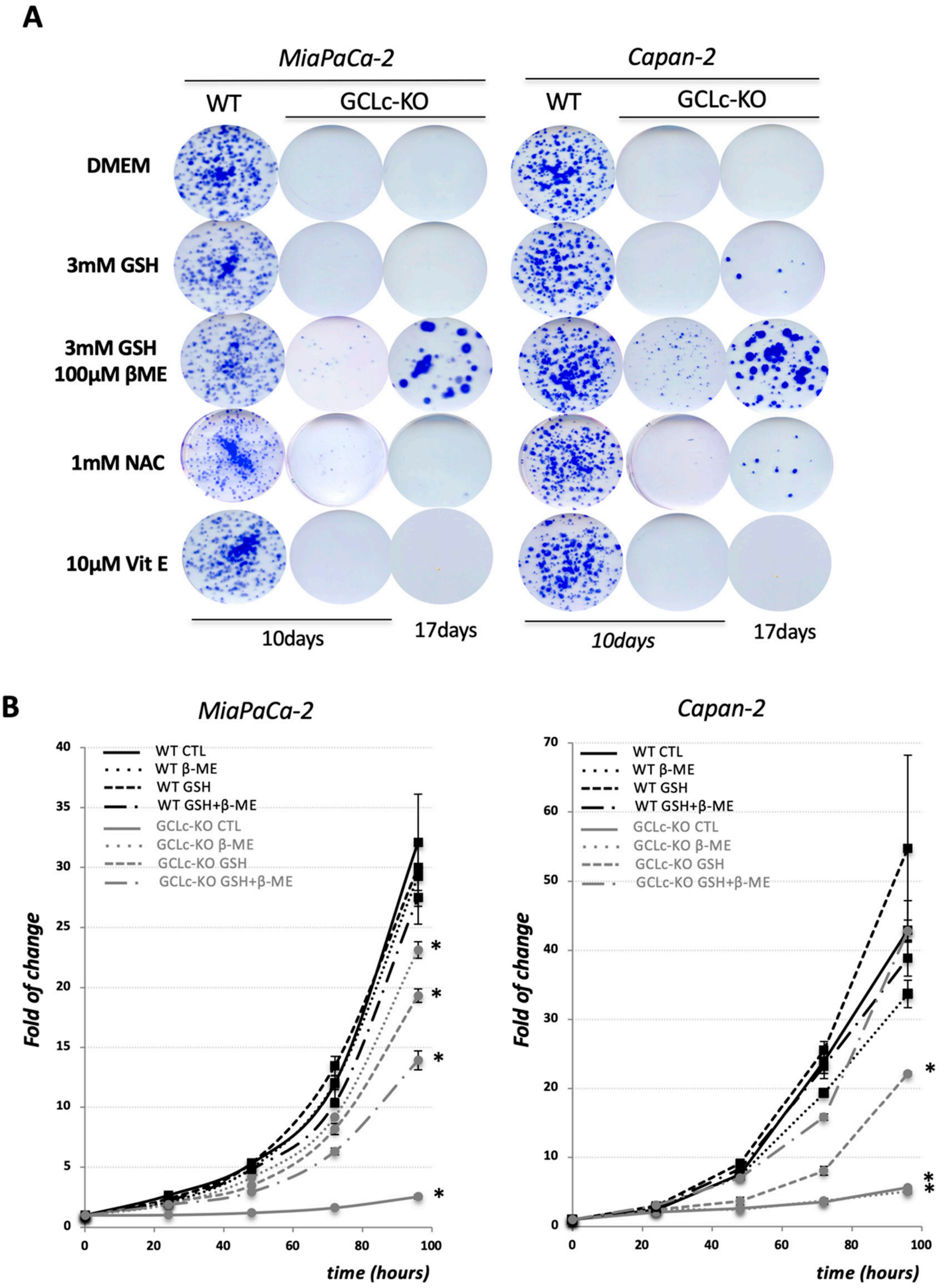

Figure S3. Proliferation and clonogenicity potential of WT and GCLc-KO cells of two PDAC cell lines. A. 10- or 17- day clonal growth of MiaPaCa-2 and Capan-2 WT and GCLc-KO cells in the

presence or not of 3mM GSH, 3mM GSH + 10 M -ME, 1mM NAC, 10 M vitamin E. Representative images are shown. B. Proliferation of MiaPaCa-2 and Capan-2 WT and GCLc-KO cells seeded in the standard DMEM media supplemented or not with 3mM GSH, 100 M -ME or their combination. Proliferation rates are presented as fold of change (mean SEM; n=3), *P < 0.05, comparison with WT control cells (presented only 96-hour time point).

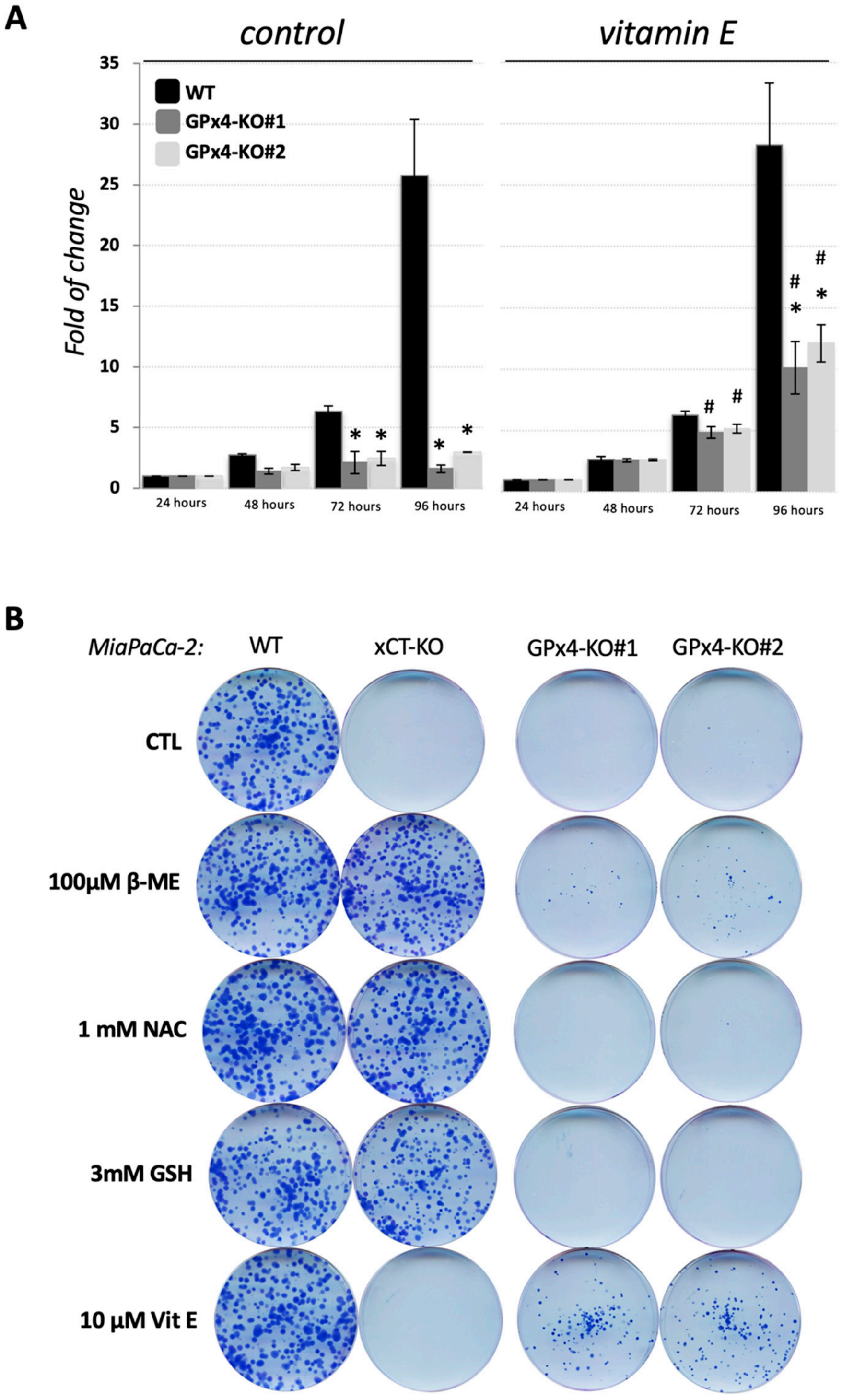

Figure S4. Proliferation and clonogenicity potential of MiaPaCa-2 WT and GPx4-KO cells. A. Proliferation of MiaPaCa-2 WT and GPx4-KO cells seeded in the standard DMEM media supplemented or not with 10 M vitamin E. Proliferation rates are presented as fold of change (mean SEM; n=3), *P < 0.05, comparison with WT control cells. #P < 0.05, comparison with corresponding cell line in control conditions. B. 10-day clonal growth of MiaPaCa-2 WT, xCT-KO and GCLc-KO cells in the presence or not of 100 M -ME, 1mM NAC, 3mM GSH, or 10 M vitamin E. Representative images are shown.

Figure 1A

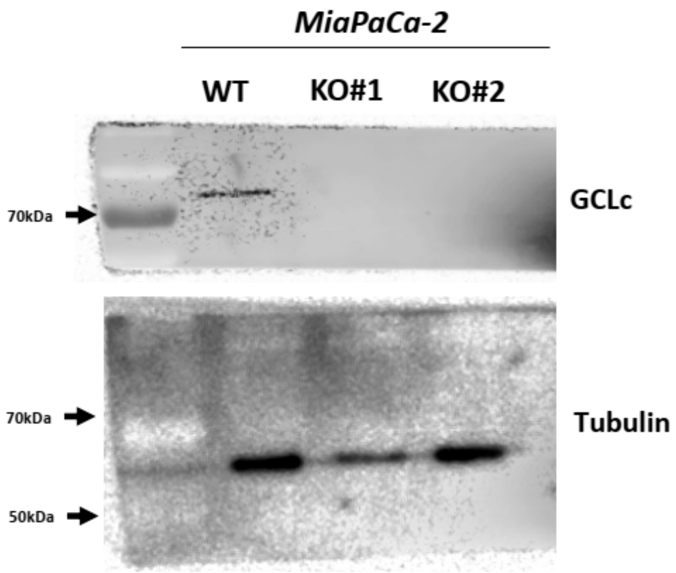

Figure 1B

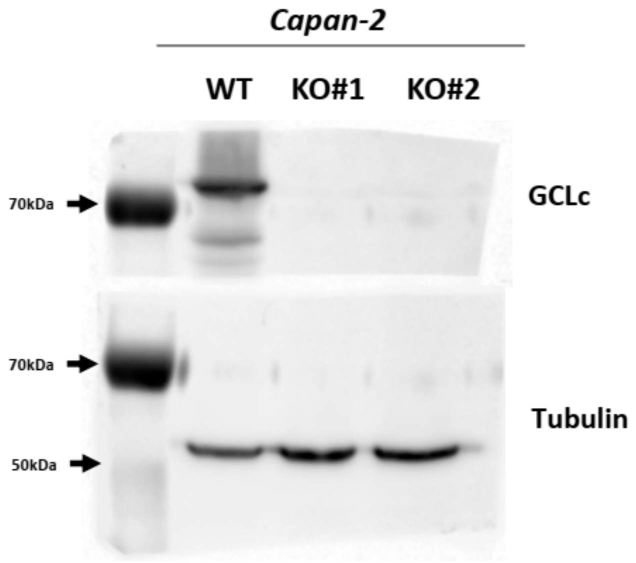

Figure 3B

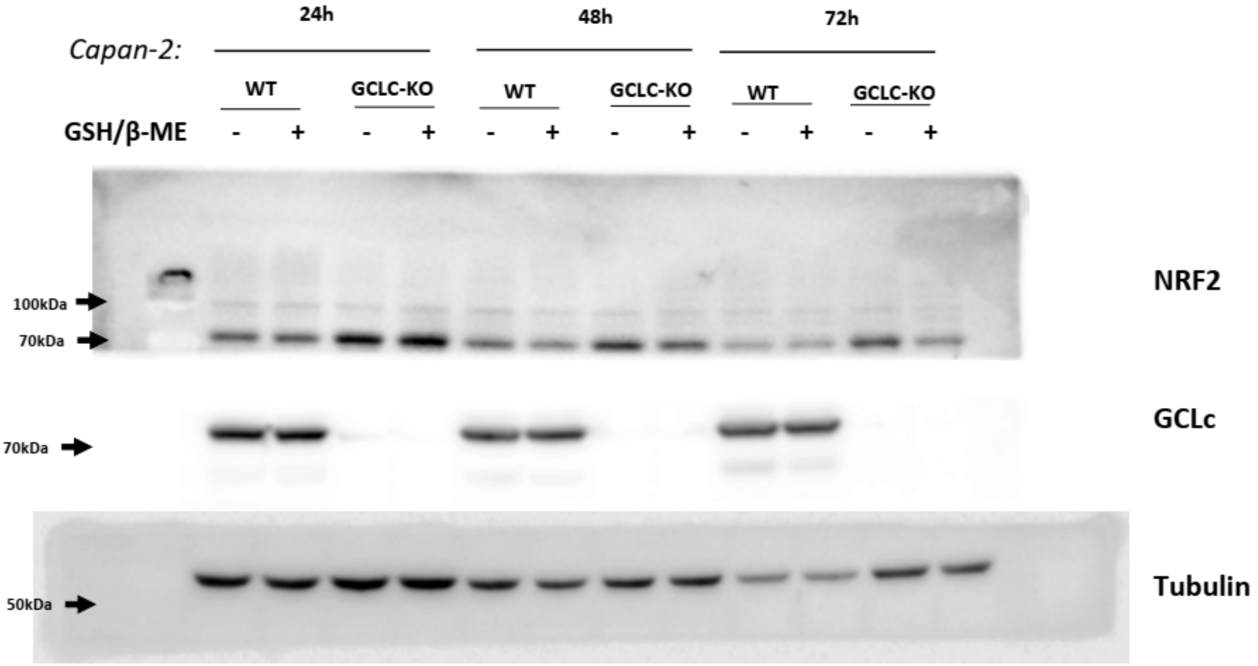

Figure 4C

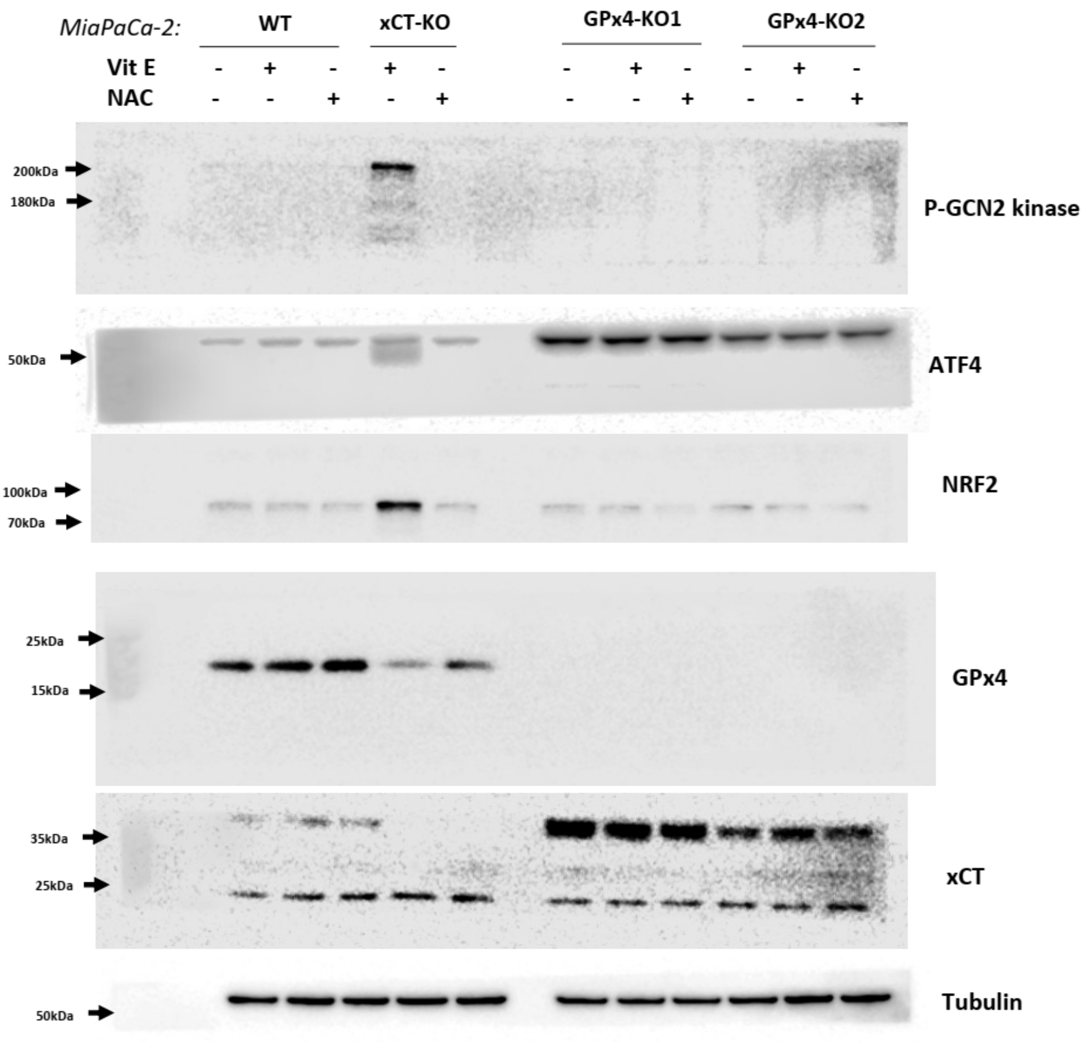

Figure 5B

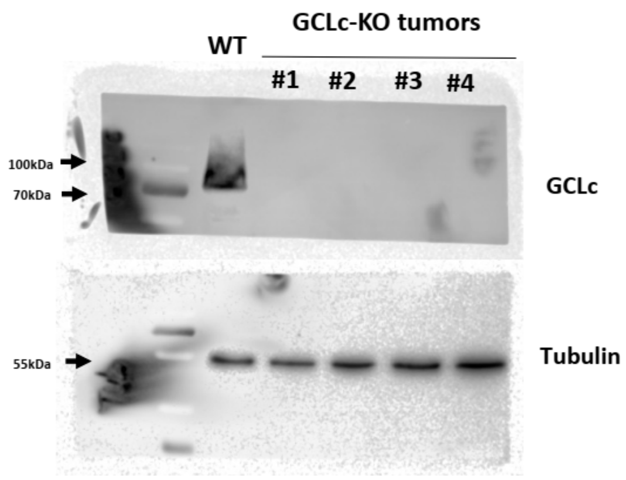

Figure S5. Original uncropped Western blots of Figure 1A and B, Figure 3B, Figure 4C, and Figure 5B.
